# ROS regulation of axonal mitochondrial transport is mediated by Ca^2+^ and JNK in *Drosophila*

**DOI:** 10.1371/journal.pone.0178105

**Published:** 2017-05-18

**Authors:** Pin-Chao Liao, Lauren C. Tandarich, Peter J. Hollenbeck

**Affiliations:** Department of Biological Sciences, Purdue University, West Lafayette, Indiana, United States of America; Texas Technical University Health Sciences Center, UNITED STATES

## Abstract

Mitochondria perform critical functions including aerobic ATP production and calcium (Ca^2+^) homeostasis, but are also a major source of reactive oxygen species (ROS) production. To maintain cellular function and survival in neurons, mitochondria are transported along axons, and accumulate in regions with high demand for their functions. Oxidative stress and abnormal mitochondrial axonal transport are associated with neurodegenerative disorders. However, we know little about the connection between these two. Using the *Drosophila* third instar larval nervous system as the *in vivo* model, we found that ROS inhibited mitochondrial axonal transport more specifically, primarily due to reduced flux and velocity, but did not affect transport of other organelles. To understand the mechanisms underlying these effects, we examined Ca^2+^ levels and the JNK (c-Jun N-terminal Kinase) pathway, which have been shown to regulate mitochondrial transport and general fast axonal transport, respectively. We found that elevated ROS increased Ca^2+^ levels, and that experimental reduction of Ca^2+^ to physiological levels rescued ROS-induced defects in mitochondrial transport in primary neuron cell cultures. In addition, *in vivo* activation of the JNK pathway reduced mitochondrial flux and velocities, while JNK knockdown partially rescued ROS-induced defects in the anterograde direction. We conclude that ROS have the capacity to regulate mitochondrial traffic, and that Ca^2+^ and JNK signaling play roles in mediating these effects. In addition to transport defects, ROS produces imbalances in mitochondrial fission-fusion and metabolic state, indicating that mitochondrial transport, fission-fusion steady state, and metabolic state are closely interrelated in the response to ROS.

## Introduction

Mitochondria perform functions that are critical for neuronal survival, such as ATP production and Ca^2+^ homeostasis. However, the mitochondrial electron transport chain is also a major source of reactive oxygen species (ROS) production [1, 2]. ROS are generated from incomplete reduction of oxygen, and include the superoxide anion (O_2_^-^), hydrogen peroxide (H_2_O_2_) and the hydroxyl radical (HO·). Under physiological conditions, ROS serve as important signaling molecules [3, 4]. However, excess ROS induce oxidative stress that harms cells by reacting with and damaging macromolecules or other subcellular structures [2, 5]. To maintain optimal cellular redox balance, cells deploy a variety of antioxidant enzymes, including superoxide dismutase (SOD), catalase, and glutathione peroxidase (GSH Px), that eliminate excess ROS [6]. In neurons, an increase in oxidative stress and/or a reduction of antioxidants has been shown to induce neurodegeneration both *in vitro* and *in vivo* [1, 7–9]. The imbalance of redox status is proposed to be a major factor or symptom of a variety of neurodegenerative diseases, such as Parkinson’s disease, Alzheimer’s disease, and amyotrophic lateral sclerosis (ALS), [7, 10].

However, oxidative damage is not the only factor that can induce neurodegenerative disease. The asymmetry and compartmentalization of neurons require that they transport mitochondria to different regions, and accumulate mitochondria at locations with demand for their functions [11]. Thus, impaired mitochondrial transport in axons has been associated with several neurodegenerative diseases [11–14]. Mitochondrial transport is regulated in part by Ca^2+^, which binds to the EF-domain of mitochondrial Rho GTPase (Miro) and changes the capacity of mitochondria to bind kinesin motor proteins via the adaptor protein Milton. Thus, elevated intracellular Ca^2+^ levels lead to reduced mitochondrial transport [15–17]. In addition to Ca^2+^ levels, several signaling pathways such as the MAPK, JNK, and Akt/GSK3β pathways have been shown to regulate axonal organelle transport [18–21]. Moreover, because elements of the mitochondrial life cycle including movement, morphology changes, biogenesis, and degradation are highly interrelated, disruption of mitochondrial fusion-fission balance also affects mitochondrial transport [22–24].

Although both ROS and impaired axonal transport of mitochondria are implicated in neurodegenerative diseases, there is little evidence about whether and how ROS directly affect mitochondrial transport. Recent studies have shown that H_2_O_2_ treatment in cell culture systems leads to the reduction of mitochondrial transport [25]. In addition, when Cu/Zn superoxide dismutase (SOD1) is mutated, mitochondrial transport decreases *in vitro* [26]. Whether these effects occur in the more complex, homeostatic, and normoxic *in vivo* environment remains unknown. Moreover, the mechanisms involved in regulating mitochondrial transport under oxidative stress conditions remain unclear.

In this study, we employed primary *Drosophila* neuronal cell culture and the third instar larval nervous system as *in vitro* and *in vivo* models, respectively, to study mitochondrial transport under oxidative stress conditions. We found that oxidative stress decreased mitochondrial axonal transport not only *in vitro* but also *in vivo*. Importantly, oxidative stress did not affect axonal transport more generally, but was more specific to mitochondria. Furthermore, we found evidence suggesting that both Ca^2+^ levels and the JNK pathway mediate the modulation of mitochondrial transport that occurs in the presence of excess ROS. These results indicate that the interaction of ROS and mitochondrial transport is not simply through non-specific damage but also through the imbalance of Ca^2+^ homeostasis and disruption of signaling.

## Results

### Mitochondrial transport in axons is reduced in response to ROS

Impairment of the axonal transport of mitochondria has been shown to result in neurodegenerative diseases [11, 14]. However, we know little about how ROS production might circle back to regulate mitochondrial transport, an issue that may be important in the pathophysiology of neurodegeneration. Although diminished overall mitochondrial transport under oxidative stress conditions has been shown in cultured neurons [25], it remains unknown whether oxidative stress affects mitochondrial transport in in the more complex *in vivo* environment, in which homeostatic control of ROS is probably more robust and physiological pO_2_ is lower.

**Fig 1:**
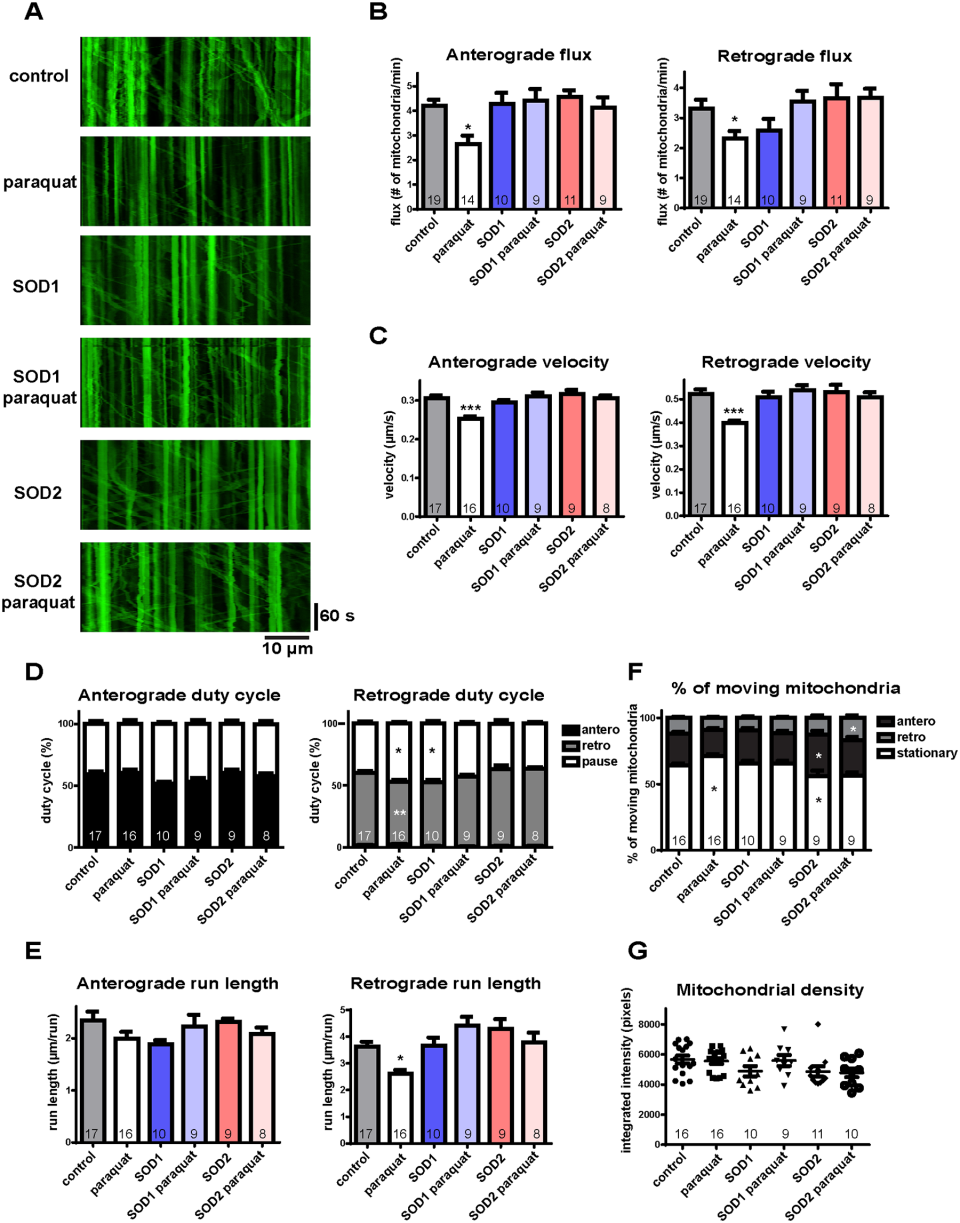
ROS changes mitochondrial motility mainly by reducing flux and velocity *in vivo*. (A) Representative kymographs of mitochondrial transport under paraquat treatment. Anterograde transport is toward the right; retrograde transport is toward the left. (B) Paraquat treatment produces a decrease in mitochondrial flux, which is rescued by overexpression of SOD1 or SOD2 in both directions. (C) Velocity is reduced with paraquat treatment, and this is rescued by SOD1 or SOD2 overexpression in both directions. (D) Paraquat treatment shows a small reduction in retrograde duty cycle with an increase of pause time and a decrease of moving time; overexpression of SOD1 shows a small increase of pause time. (E) Retrograde run length is modestly reduced with paraquat treatment. (F) Paraquat treatment shows an increase of the percentage of stationary mitochondria, which is rescued by SOD overexpression. The percentage of anterograde moving mitochondria increases with SOD2 overexpression. (G) Mitochondrial density is comparable to control with paraquat treatment or SOD overexpression. The number of larvae analyzed is shown on the bars. Error bars indicate mean ± SEM. Significance is determined by one-way ANOVA with Bonferroni’s post-test. *p<0.05, **p < 0.01, and ***p < 0.001.

To test whether ROS affect mitochondrial transport in axons *in vivo*, we quantified all parameters of mitochondrial transport in 3rd instar *Drosphila* larvae expressing mito-GFP in motor neurons and treated with 20 mM paraquat for 24 hrs [27]. Using larval preparation as we have previously described (Shidara et al, 2010; Devireddy et al, 2014), we observed mitochondrial traffic in axons still connected to their cell bodies and synapses in the central nervous system. In axons, moving mitochondria are categorized into anterograde and retrograde populations by their dominant directions, which are easily discernible despite pauses and brief reversals of direction (Fig 1A). We first quantified flux, which is a gross indicator of movement representing how many mitochondria pass a fixed point per unit time. We found reduced organelle flux in both the anterograde and retrograde populations after paraquat treatment (Fig 1B). To parse this effect further and determine the possible components producing the reduced flux, we examined more specific parameters of mitochondrial motility: velocity, duty cycle, run length, percentage of moving mitochondria, and density [28]. Under oxidative stress conditions, we found reduced instantaneous velocity in both directions (Fig 1C). In addition, we found a modestly-reduced duty cycle in the retrograde direction (Fig 1D), indicating that retrograde mitochondria spent less time moving in the dominant direction. Retrograde run length was also modestly reduced, perhaps as a consequence of the reduced velocity and duty cycle (Fig 1E). Moreover, an increased percentage of stationary mitochondria (Fig 1F) also contributed to the reduced flux. Mitochondrial density is not necessarily directly linked to motility, but reduced density can of course contribute to reduced flux [28]. However, we found no change in the mitochondrial density of segmental nerve axons under oxidative stress conditions (Fig 1G), indicating that mitochondrial motility itself is reduced under these conditions *in vivo*.

### Overexpression of SOD attenuates ROS-induced defects of mitochondrial transport

To further ensure that this decreased mobility was specifically caused by oxidative stress, we took advantage of the observation that SOD overexpression has been shown to relieve oxidative stress in *Drosophila* [29, 30]. We found that the reduced mitochondrial flux and velocity in both directions produced by paraquat treatment were rescued by SOD1 or SOD2 overexpression (Fig 1B and 1C). In the retrograde direction, both reduced duty cycle and run length were also rescued (Fig 1D and 1E). In addition, the defects in the percentage of moving mitochondria produced in response to ROS were fully rescued by SOD1 or SOD2 overexpression (Fig 1F). These results indicate that a reduction of the excess ROS levels can diminish or eliminate the defects in mitochondrial transport. On the other hand, the axonal mitochondrial density was comparable to controls in larva with SOD1 or SOD2 overexpression alone (Fig 1G), suggesting that mitochondrial biogenesis and/or the anterograde-retrograde traffic steady state are not affected by the overexpression. Altogether, these results indicate that excess ROS do cause specific defects in the axonal transport of mitochondria *in vivo*.

### Transport of neuropeptide-bearing large dense core vesicles (DCVs) is barely affected by the presence of ROS

**Fig 2:**
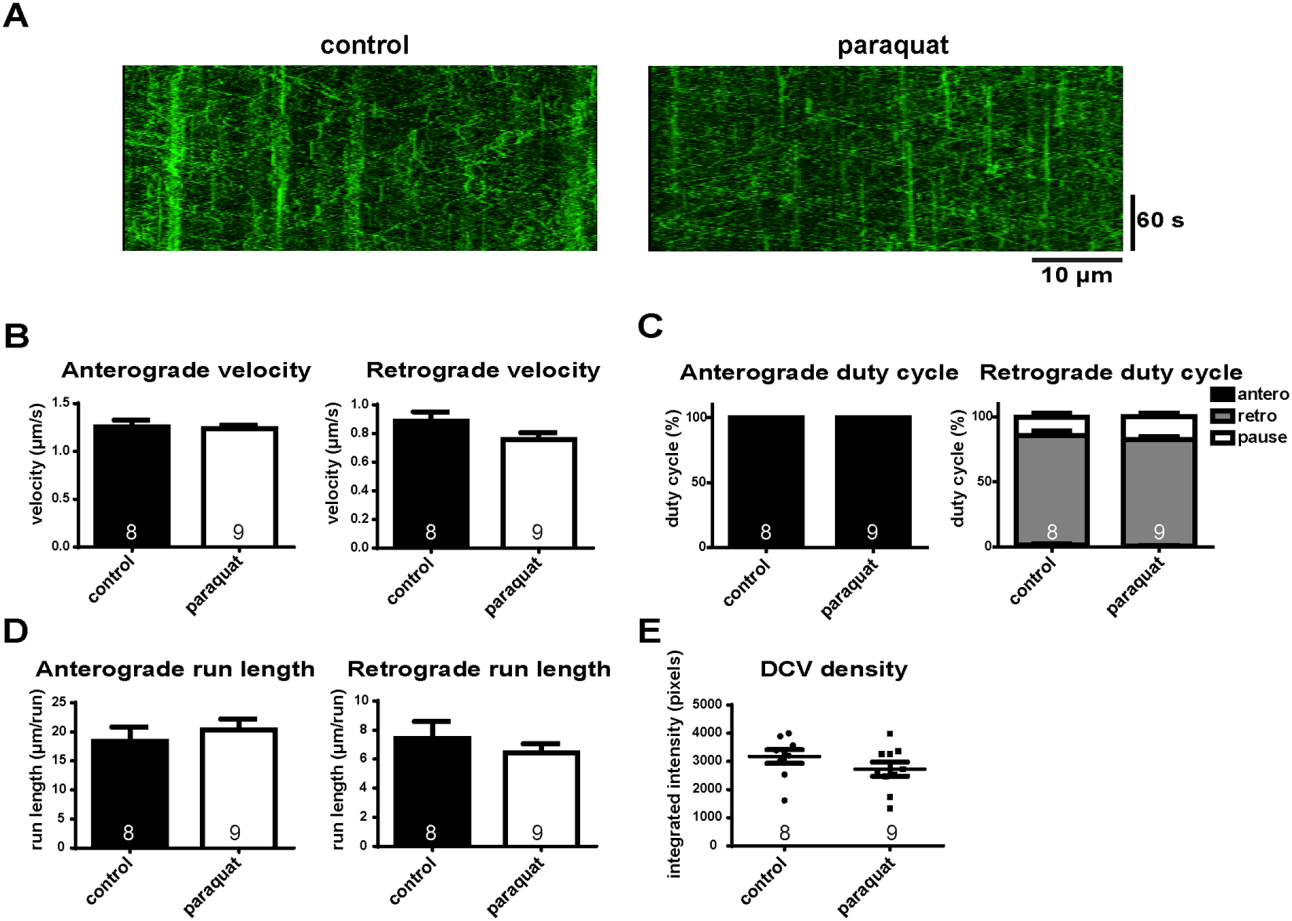
DCV transport is nearly unaffected by ROS treatment. (A) Representative kymographs of DCV transport *in vivo*. Anterograde transport is toward the right; retrograde transport is toward the left. Parameters in DCV transport with paraquat treatment including (B) velocity, (C) duty cycle, (D) run length, and (E) density are all comparable to controls. The number of larvae analyzed is shown on the bars. Error bars indicate mean ± SEM. Significance is determined by Student’s t-test.

To test whether the defects of transport in response to ROS are specific to mitochondria, we expressed ANF-GFP, targeted to the lumen of neuropeptide-bearing large DCVs, in motor neurons [31], and examined their transport under oxidative stress conditions. Detailed parameters of DCV transport, including velocity, duty cycle, run length, and density were analyzed (Fig 2). All parameters of DCV transport in response to ROS were comparable to controls (Fig 2B–2D), indicating that transport of DCVs is barely affected by oxidative stress. In addition, the axonal DCV density was similar to controls (Fig 2E), implying an unchanged steady state of DCV biogenesis, transport, and degradation. Altogether, these data indicate that oxidative stress impedes mitochondrial transport more specifically, rather than the general axonal transport of organelles.

### ROS reduce mitochondrial transport by the elevation of Ca^2+^ levels

Elevated intracellular Ca^2+^ levels decrease mitochondrial transport by binding the EF domain on Miro [15–17]. In addition, since there are significant interactions between Ca^2+^ and redox signaling [32–34], we asked whether the defects in mitochondrial transport caused by oxidative stress might be mediated by the elevation of intracellular Ca^2+^ levels. To test this, we used primary neuronal cells as our system since experimental manipulation of Ca^2+^ levels *in vivo* is difficult.

**Fig 3:**
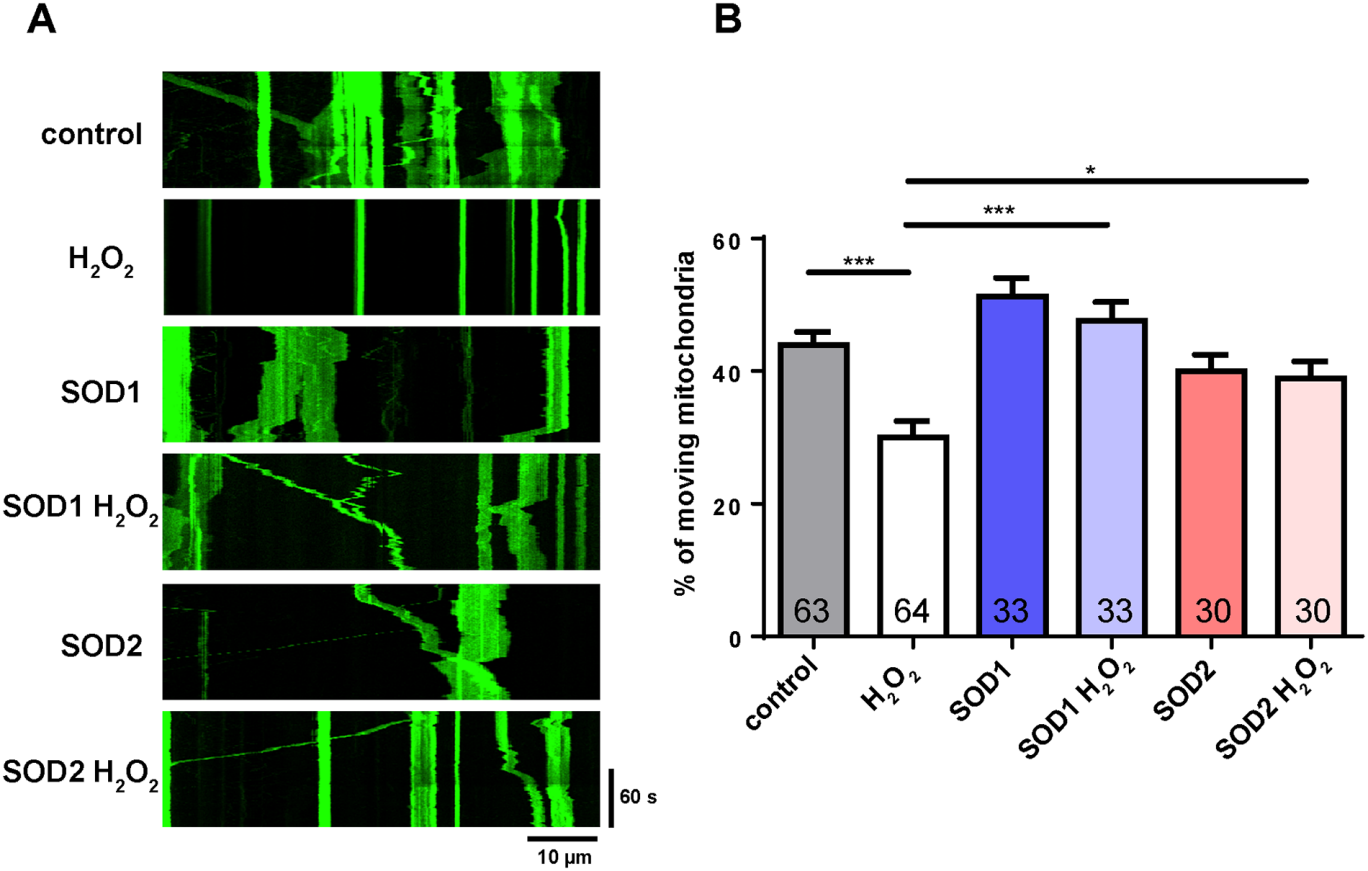
The percentage of moving mitochondria is reduced in response to ROS and rescued by SOD overexpression *in vitro*. (A) Representative kymographs of mitochondrial transport under H_2_O_2_ treatment. Anterograde transport is toward the right; retrograde transport is toward the left. (B) The percentage of moving mitochondria is reduced with H_2_O_2_ treatment. SOD1 or SOD2 overexpression can rescue the defect. The number of cells analyzed is shown on the bars. Error bars indicate mean ± SEM. Significance is determined by one-way ANOVA with Bonferroni’s post-test. *p<0.05 and ***p < 0.001.

We first ensured that mitochondrial transport was also reduced in response to ROS in our *in vitro* system in *Drosophila*. To assess this, primary neuron cell cultures obtained from brains and ventral ganglia of third instar larvae expressing mitochondrially-targeted GFP in motor neurons were treated with 100 μM H_2_O_2_ for 1 hr. In control cells, ~40% of mitochondria were mobile, whereas the percentage of moving mitochondria in H_2_O_2_-treated cells decreased significantly (Fig 3). Interestingly, mitochondrial ROS did not show significant increase after 1 hr using ROS indicators roGFP expressed in mitochondria. Instead, mitochondrial ROS levels did increase after 2 hrs, but the motility defects did not become worse (S1 Fig). These data show that mitochondrial ROS are not required to induce motility defects, suggesting that cytosolic ROS are sufficient to induce motility defects. In addition, similar to the results *in vivo* (Fig 1), the defects in the percentage of moving mitochondria produced in response to ROS were fully rescued by SOD1 or SOD2 overexpression (Fig 3), indicating that oxidative stress impair mitochondrial transport both *in vitro* and *in vivo*.

**Fig 4:**
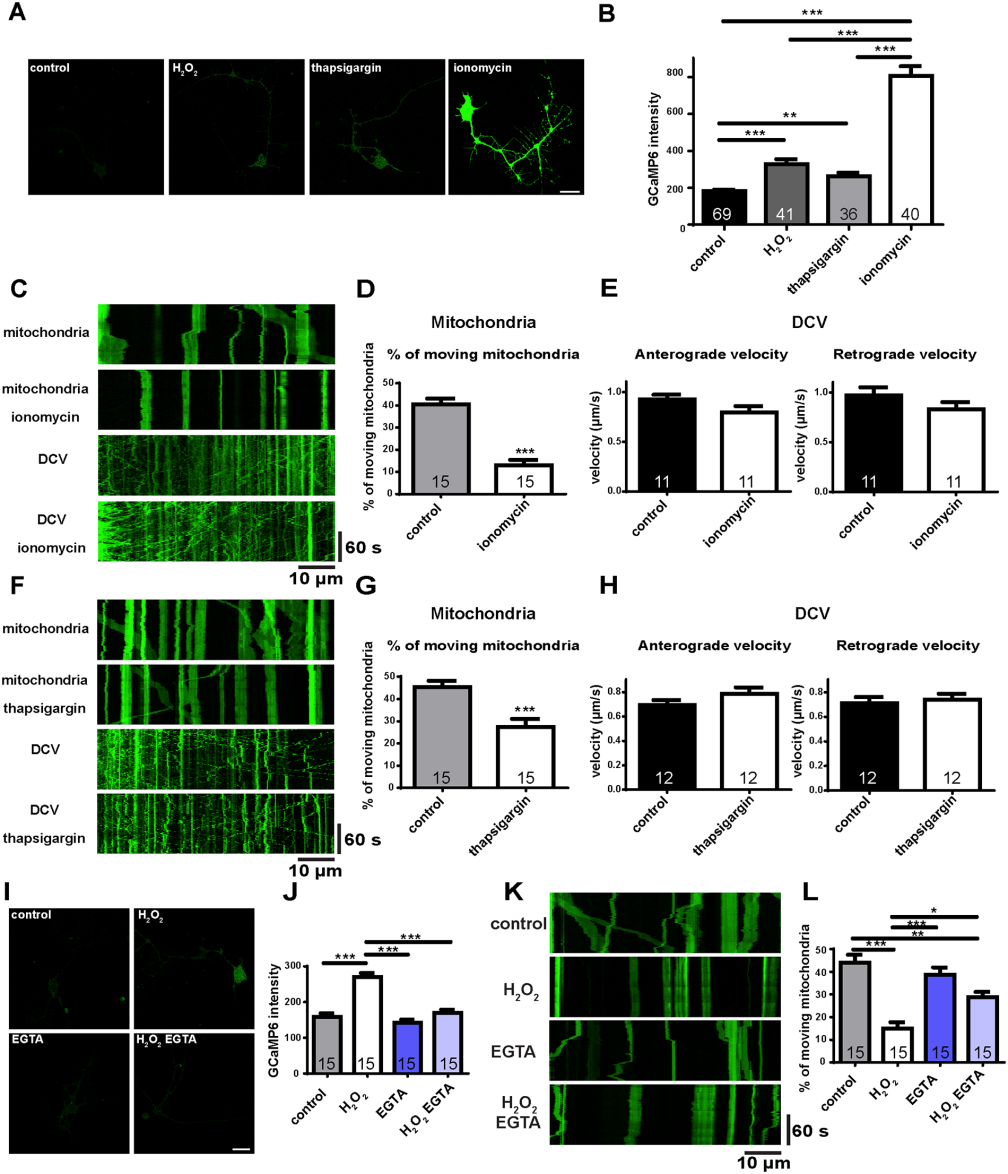
ROS-induced defects in mitochondrial transport are mediated by Ca^2+^ levels. (A) Representative Ca^2+^ imaging with H_2_O_2_, thapsgargin, or ionomycin treatment is measured by the intensity of GCaMP6 indicator. Scale bars indicate 10 μm. (B) Quantitative results from (A). H_2_O_2_ or thapsgargin treatment produces an increase of Ca^2+^ levels of similar extent. Ionomycin treatment produces a large increase compared to controls. (C) Representative kymographs of axonal transport of mitochondria or DCVs before and after ionomycin treatment in the same cell. Anterograde transport is toward the right; retrograde transport is toward the left. (D) The percentage of moving mitochondria is dramatically reduced by ionomycin treatment. (E) Velocity of DCV transport is not affected by ionomycin. (F) Representative kymographs of axonal transport of mitochondria or DCVs before and after thapsigargin treatment in the same cell. (G) The percentage of moving mitochondria is reduced by thapsigargin treatment, but the effect is not as large as ionomycin treatment. (H) Velocity of DCV transport is not affected by thapsigargin. (I) Representative Ca^2+^ imaging with H_2_O_2_ or EGTA treatment is measured by the intensity of GCaMP6 indicator. Scale bars indicate 10 μm. (J) Quantitative results from (I). Elevated Ca^2+^ levels induced by H_2_O_2_ are rescued by EGTA treatment. EGTA alone does not affect intracellular Ca^2+^ levels. (K) Representative kymographs of mitochondrial transport under H_2_O_2_ or EGTA treatment. Anterograde transport is toward the right; retrograde transport is toward the left. (L) The reduced percentage of moving mitochondria by H_2_O_2_ is partially rescued by EGTA. EGTA alone does not affect mitochondrial transport. The number of cells analyzed is shown on the bars. Error bars indicate mean ± SEM. Significance is determined by one-way ANOVA with Bonferroni’s post-test (B, J, L) or paired Student’s t-test (D, E, G, H). *p<0.05, **p < 0.01, and ***p < 0.001.

Next, we expressed the genetically encoded Ca^2+^ indicator GCaMP6 in motor neurons using the *D42-Gal4* driver and determined the response of Ca^2+^ levels to ROS [35]. We found that cells with H_2_O_2_ treatment for 1 hr showed about 50% higher GCaMP6 intensity compared to controls (Fig 4A and 4B), indicating that excess ROS can induce elevated intracellular Ca^2+^ levels. We then examined whether elevated Ca^2+^ levels decrease mitochondrial transport and/or affect DCV transport. We treated cells with the Ca^2+^ ionophore, ionomycin, which produced a dramatic increase in Ca^2+^ levels (Fig 4A and 4B), and examined both mitochondrial transport and DCV transport (Fig 4C). Notably, dramatically increased Ca^2+^ levels only affected mitochondrial transport (Fig 4C and 4D). When we examined DCV transport, we found no significant difference in velocity of DCV transport between control and ionomycin-treated cells (Fig 4C and 4E). Although mitochondrial transport was reduced in ionomycin-treated cells, Ca^2+^ levels were much higher than physiological or ROS-treated levels. Thus, we used thapsigargin, an ER Ca^2+^ ATPase inhibitor that increases only intracellular Ca^2+^ levels, to mimic Ca^2+^ levels in response to ROS (Fig 4A and 4B). Under these conditions, the percentage of moving mitochondria decreased to a level similar to H_2_O_2_-treated cells (Fig 4F and 4G), and velocity of DCV transport was comparable to controls (Fig 4F and 4H). Thus, elevated Ca^2+^ levels reduced mitochondrial transport, as expected [15–17] and did so more specifically.

These data (Fig 4A–4H) support the hypothesis that ROS decrease mitochondrial transport via the elevation of Ca^2+^ levels. To test this directly we treated cells with Ca^2+^-free medium containing EGTA and exposed them to H_2_O_2_, to examine whether the defects of mitochondrial transport caused by ROS would be rescued. First we confirmed that the elevated Ca^2+^ levels caused by H_2_O_2_ treatment were reduced in Ca^2+^-free medium with EGTA using the GCaMP6 indicator (Fig 4I and 4J). Next, we examined mitochondrial transport under these conditions. In the Ca^2+^-free medium containing EGTA, there was no increase of the percentage of moving mitochondria above normal (Fig 4K and 4L) since the Ca^2+^ levels are comparable to controls (Fig 4I and 4J). Moreover, under ROS treatment with Ca^2+^ held to control levels, the ROS-induced reduction of moving mitochondria was partially rescued (Fig 4K and 4L), indicating that ROS-induced defects of mitochondrial transport are mediated in large part by the elevation of Ca^2+^ levels.

**Fig 5:**
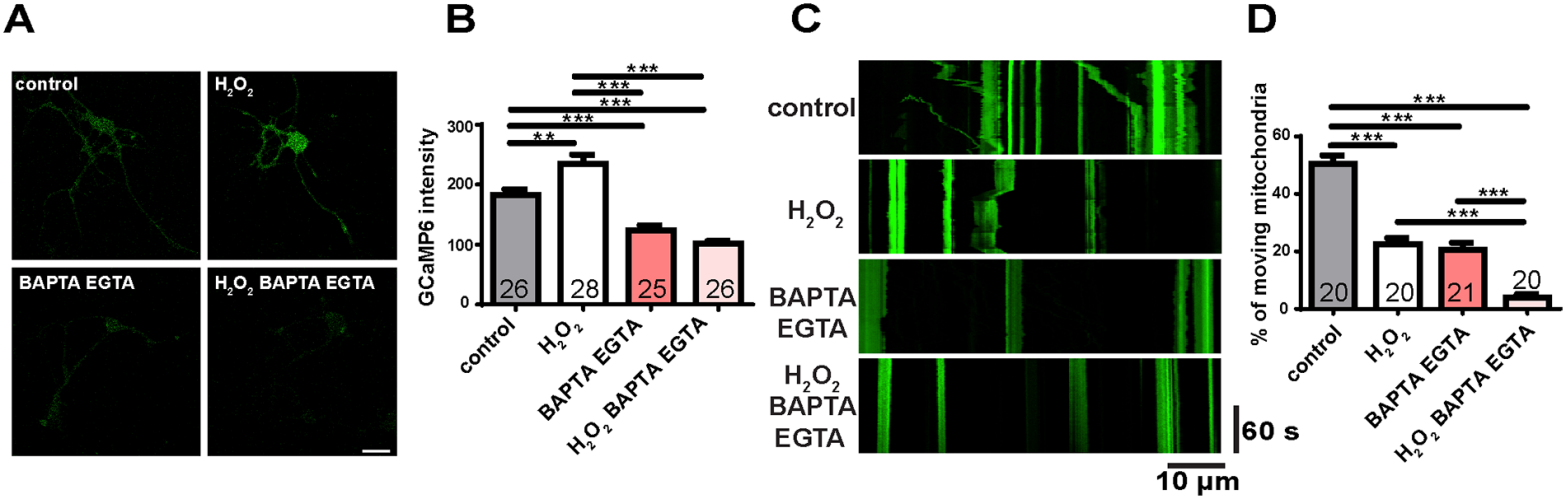
Ca^2+^ homeostasis is required for normal mitochondrial transport. (A) Representative Ca^2+^ imaging with H_2_O_2_ or EGTA/BAPTA treatment is measured by the intensity of GCaMP6 indicator. Scale bars indicate 10 μm. (B) Quantitative results from (A). Ca^2+^ levels are increased by H_2_O_2_ but reduced with EGTA/BAPTA treatment. (C) Representative kymographs of mitochondrial transport with H_2_O_2_ or EGTA/BAPTA treatment. Anterograde transport is toward the right; retrograde transport is toward the left. (D) EGTA/BAPTA treatment produces a decrease of mitochondrial transport. The reduced percentage of moving mitochondria by H_2_O_2_ is further reduced by EGTA/BAPTA treatment. The number of cells analyzed is shown on the bars. Significance is determined by one-way ANOVA with Bonferroni’s post-test. **p < 0.01, and ***p < 0.001.

Since EGTA treatment, rescuing Ca^2+^ to physiological levels, rescued ROS-induced defects of mitochondrial transport, we wondered whether further reduction of Ca^2+^ levels could increase mitochondrial transport. To examine this, we first treated cells with BAPTA-AM, which is a Ca^2+^ chelator that can cross the cell membrane. However, we verified that the BAPTA-AM only was not enough to chelate both extracellular and intracellular Ca^2+^ by treating cells with BAPTA and ionomycin (S2 Fig). Therefore, we treated both BAPTA and EGTA to chelate both extracellular and intracellular Ca^2+^. Under these conditions, intracellular Ca^2+^ levels were reduced compared to controls (Fig 5A and 5B). In addition, under oxidative stress conditions, Ca^2+^ levels decreased in the Ca^2+^-free medium containing BAPTA-AM and EGTA (Fig 5C and 5D), indicating that combined intracellular and extracellular Ca^2+^ chelation indeed reduces Ca^2+^ levels lower than physiological levels. Surprisingly, mitochondrial transport was decreased by treatment with both BAPTA-AM and EGTA (Fig 5C and 5D), indicating that Ca^2+^ levels that are below physiological levels can also inhibit mitochondrial transport. All together, these data suggest that modulation of Ca^2+^ levels mediates the effect of ROS on mitochondria, and emphasize that Ca^2+^ homeostasis is important for mitochondrial transport.

### The JNK pathway plays a role in the regulation of mitochondrial transport in axons by oxidative stress

The involvement of Ca^2+^ suggests that the effects of ROS exposures on mitochondrial transport probably reflect not just generalized oxidative damage, but a disturbance of normal ROS-based signaling. To investigate this, we examined the JNK pathway, since it is both activated under oxidative stress conditions [6, 36] and involved in regulating axonal transport [20, 37]. Previous studies have shown that activation of the JNK pathway causes phosphorylation of kinesin heavy chain (Khc), which causes the kinesin motor to release from microtubules, and thus reduces anterograde axonal transport in squid axoplasm [20]. We tested the hypothesis that under oxidative stress conditions anterograde mitochondrial transport is regulated by the JNK pathway.

**Fig 6:**
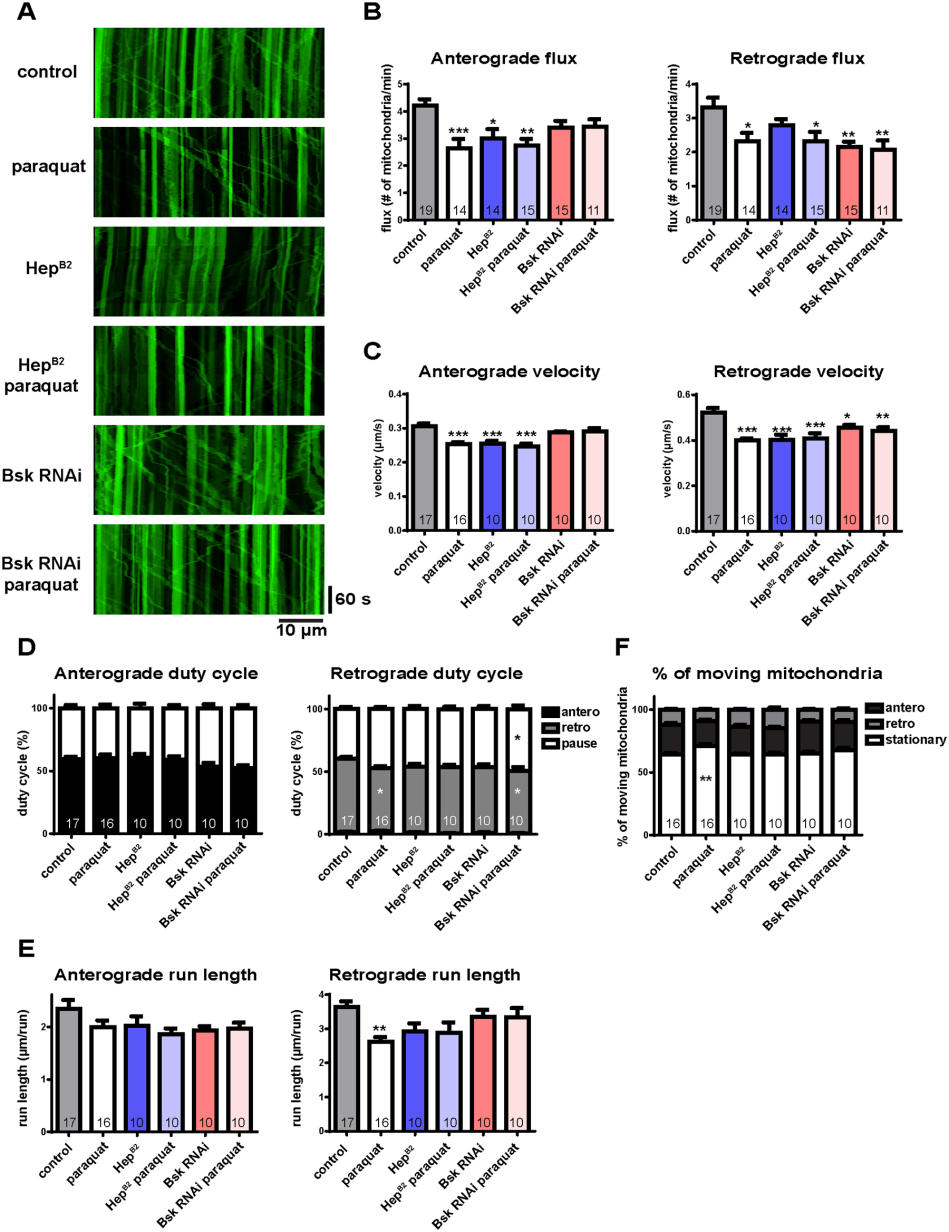
The JNK pathway plays a role in the regulation of mitochondrial transport *in vivo*. (A) Representative kymographs of mitochondrial transport with overexpression of JNK kinase (Hep^B2^) or knockdown of JNK (Bsk RNAi) in response to paraquat. Anterograde transport is toward the right; retrograde transport is toward the left. (B) Paraquat and/or overexpression of Hep^B2^ produce a decrease in mitochondrial flux anterogradly. Knockdown of Bsk partially rescues the effect of paraquat. Both overexpression of Hep^B2^ and knockdown of Bsk reduce retrograde flux. (C) Anterograde velocity is reduced by paraquat or overexpression of Hep^B2^, while retrograde velocity is reduced in both overexpression of Hep^B2^ and knockdown of Bsk. (D) Paraquat treatment shows slightly reduction in retrograde duty cycle with a decrease of moving time; Knockdown of Bsk in response to paraquat shows a slightly increase of pause and a decrease of moving time. (E) Retrograde run length is modestly reduced by paraquat treatment. Either overexpression of Hep^B2^ of knockdown of Bsk does not show significant difference compared to controls. (F) Paraquat treatment shows an increase of the percentage of stationary mitochondria, while neither overexpression of Hep^B2^ nor knockdown of Bsk shows any difference. The number of larvae analyzed is shown on the bars. Error bars indicate mean ± SEM. Significance is determined by one-way ANOVA with Bonferroni’s post-test. *p<0.05, **p < 0.01, and ***p < 0.001.

To examine this, we activated the JNK pathway by overexpressing Hep^B2^ (JNK kinase in *Drosophila*), or down-regulated it by RNAi knockdown of Bsk (JNK in *Drosophila*). We expected that activation of the JNK pathway would decrease mitochondrial transport and down-regulation of the JNK pathway would increase it. Indeed, we found that anterograde flux was reduced by JNK activation. In addition, flux was not further reduced in the presence of JNK activation by the addition of oxidative stress (Fig 6A and 6B), which implicates the JNK pathway as a downstream effector of the influence of oxidative stress on anterograde mitochondrial flux. We also found that the defects in anterograde flux produced by paraquat treatment were partially rescued by JNK knockdown (Fig 6A and 6B). These data suggested that the JNK pathway plays a role, albeit not exclusive, in mediating the inhibition of mitochondrial anterograde transport by oxidative stress. Activation of the JNK pathway produced reduced anterograde velocity (Fig 6A and 6C), and down-regulation of JNK partially rescued the reduced anterograde velocity that occurred in response to ROS (Fig 6A and 6C), suggesting altered organelle velocity is one of the factors contributing to the difference in flux. For retrograde movement, either activation or down-regulation of the JNK pathway showed reduced mitochondrial flux and velocity under oxidative stress conditions (Fig 6A–6C), suggesting that other regulatory systems might be responsible for the effects of oxidative stress on retrograde traffic. While the regulatory mechanisms are directionally different, optimal levels of activity in the JNK pathway are nonetheless essential for both. Other parameters of mitochondrial motility such as duty cycles, run length, and % of moving mitochondria were only modestly affected by either overexpression or knockdown of the JNK pathway (Fig 6D–6F). Altogether, our results demonstrate that the JNK pathway, activated by oxidative stress, is involved in the regulation of mitochondrial transport in the anterograde direction, consistent with direction-specific regulation of mitochondrial transport.

Since ROS-induced defects of mitochondrial transport are mediated by Ca^2+^ and the JNK pathway, we further determined whether Ca^2+^ and the JNK pathway have interaction in the ROS regulation of mitochondrial transport. However, neither activation nor down-regulation of the JNK pathway affects intracellular Ca^2+^ levels (S3 Fig), indicating that the JNK pathway is not upstream of the Ca^2+^ signaling. This suggests that the defects of mitochondrial transport caused by ROS-induced Ca^2+^ levels are not mediated by the JNK pathway, and also raises the possibility that Ca^2+^ and JNK signaling regulate mitochondrial transport in parallel.

### Mitochondrial fission-fusion balance, inner membrane potential, and transport have interrelated response to ROS

**Fig 7:**
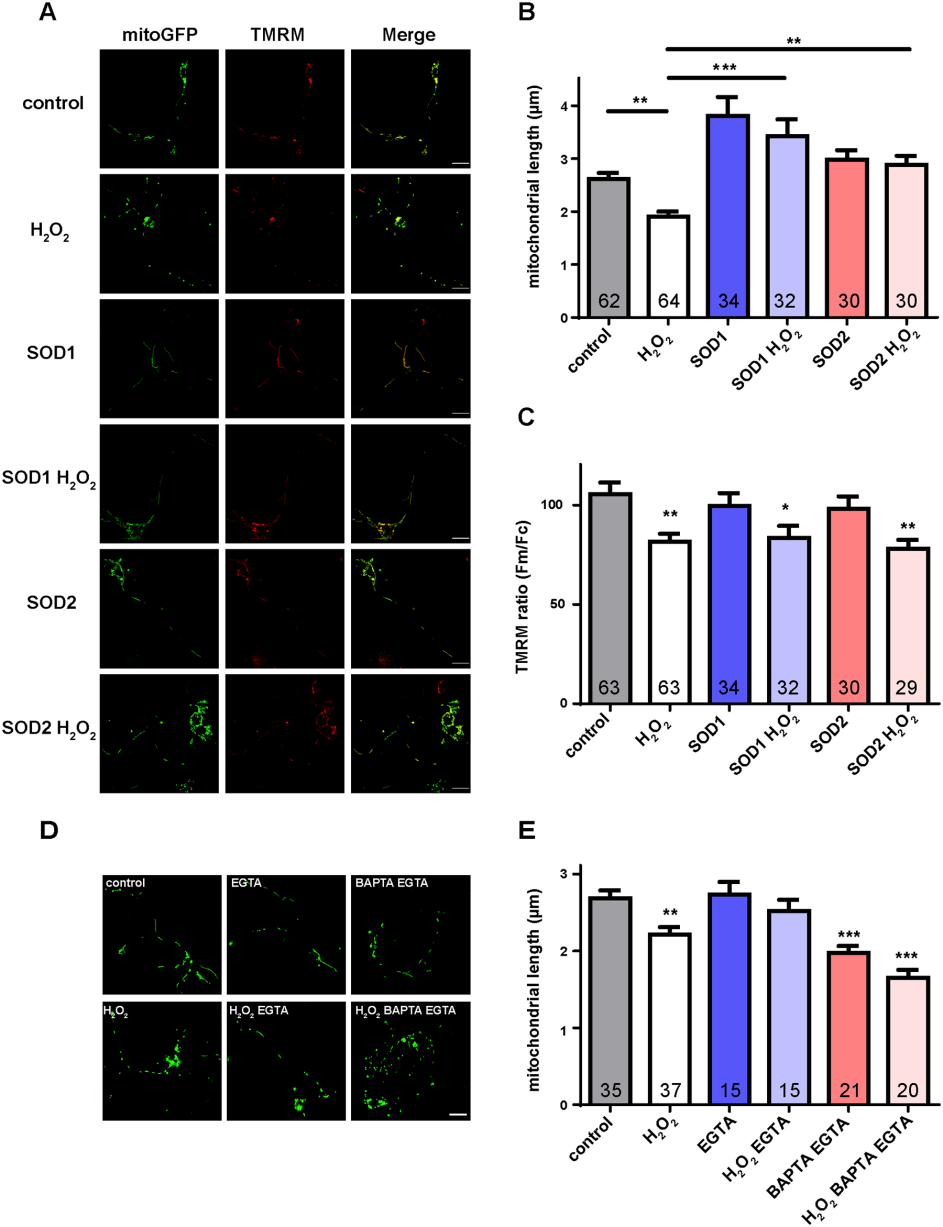
Mitochondrial length, membrane potential, and transport are interrelated in response to ROS. (A) Representative images of mitochondrial length and membrane potential. Mitochondrial lengths are measured using mitoGFP signals and mitochondrial membrane potential is measured using TMRM staining by the intensity ratio of mitochondrial fluorescence to cytosolic fluorescence. Scale bars indicate 10 μm. (B) Quantitative results of mitochondrial length. ROS treatment shows a decrease of mitochondrial length, which is rescued by SOD1 or SOD2 overexpression. (C) Quantitative results of mitochondrial membrane potential. Mitochondrial membrane potential is reduced under oxidative stress conditions. SOD1 or SOD2 overexpression does not rescue these defects. (D) Representative images of mitochondrial length measured using the mitoGFP signal. Scale bars indicate 10 μm. (E) H_2_O_2_ and/or EGTA/BAPTA reduce mitochondrial length, which is consistent with the results of mitochondrial transport (Figs 4K, 4L, 5C and 5D). The number of cells analyzed is shown on the bars. Error bars indicate mean ± SEM. Significance is determined by one-way ANOVA with Bonferroni’s post-test. *p<0.05, **p < 0.01, and ***p < 0.001.

The different elements of the mitochondrial life cycle are closely interrelated, and conditions that compromise mitochondrial motility can also alter the mitochondrial metabolic state, diminishing mitochondrial inner membrane potential [11, 38]. In turn, disruption of the fission-fusion steady-state by mutation of the mitochondrial fusion protein, mitofusin 2, induces mitochondrial fragmentation as well as decreased mitochondrial transport [22, 23, 39, 40]. To determine whether the reduced mitochondrial transport produced by ROS is accompanied by effects on metabolic state and/or fission-fusion balance, we examined both mitochondrial morphology and TMRM uptake under oxidative stress conditions *in vitro*. We found that after 1 hr of H_2_O_2_ treatment, mitochondrial shapes became more rounded and their lengths were reduced (Fig 7A and 7B). SOD1 or SOD2 overexpression rescued these defects (Fig 7A and 7B), confirming that they are induced by oxidative stress. Mitochondrial membrane potential was assessed using the intensity ratio of TMRM staining between the mitochondria and neighboring cytosol [12, 13, 28, 41]. We found reduced mitochondrial membrane potential in response to H_2_O_2_ (Fig 7A and 7C), indicating that mitochondrial metabolic function is disrupted under oxidative stress conditions. However, the diminished mitochondrial membrane potential was not rescued by SOD1 or 2 overexpression (Fig 7A and 7C), suggesting that excess SOD is not sufficient to rescue all mitochondrial defects produced by oxidative stress. Since the main function of SOD is convert O_2_^-^ to H_2_O_2_ [6], other cellular or mitochondrial ROS scavengers such as catalase or GSH Px might be required to fully rescue these defects. These data indicate that oxidative stress disrupts the mitochondrial fission-fusion steady state and metabolic function and, furthermore, that mitochondrial fission-fusion balance, membrane potential, and mobility have closely interrelated responses to oxidative stress.

Since modulation of JNK signaling and Ca^2+^ levels can mediate the effects of ROS on mitochondrial transport, we further examined whether JNK or Ca^2+^-modulated mitochondrial transport are also related to mitochondrial length control or membrane potential. Although JNK signaling had mild effects on mitochondrial length and membrane potential (S4 Fig), we found that ROS-induced mitochondrial length changes were rescued by EGTA treatment but were not rescued by both BAPTA-AM and EGTA treatment (Fig 7D and 8E), which was consistent with the transport data (Figs 4K, 4L, 5C and 5D), suggesting that mitochondrial fission-fusion steady state is closely interrelated to transport. Moreover, these data also indicate that Ca^2+^ homeostasis is important for both mitochondrial transport and mitochondrial morphology.

## Discussion

While excess ROS produce varied damage to cells [1, 2], at physiological levels they are important signaling molecules [3, 4]. ROS are produced by mitochondria [2, 10, 42] and also impact them: since they affect mitochondrial transport and quality control processes *in vitro* [25, 43–45], it is important to find underlying mechanisms among these effects. In this study, we have probed the relationship between ROS and mitochondrial transport, and have demonstrated mechanistic connections between these two. We find that: 1) ROS preferentially impair mitochondrial transport, rather than the general axonal transport of organelles; 2) ROS increase neuronal Ca^2+^ levels, and subsequently inhibit mitochondrial transport; 3) The JNK pathway plays a role in the regulation of anterograde transport in response to ROS; 4) Mitochondrial fission-fusion balance, membrane potential, and mobility have closely interrelated responses to oxidative stress.

We first found that mitochondrial axonal transport is reduced in response to ROS *in vivo*, mainly due to reduced velocity (Fig 1). More important, ROS have a more specific effect on mitochondria, as transport of DCVs is barely affected (Fig 2). This is consistent with a previous *in vitro* study [25], and indicates that mitochondria are a prime target of ROS among the axonal transport cargoes.

What could explain the specific sensitivity of mitochondrial transport to ROS? Unlike axonal vesicles, mitochondria are linked to kinesin motor proteins via a complex of Miro and Milton. Ca^2+^ binds to the two EF-domains of Miro, causing a conformational change that disrupts the ability of kinesin to move mitochondria, but interestingly, inhibiting both directions of mitochondrial transport [15–17]. Thus, Ca^2+^ regulation is a major difference in the axonal transport of mitochondria versus other organelles. The JNK pathway might also affect mitochondrial transport more specifically. Some studies indicate that the JNK pathway affects axonal transport generally via phosphorylation of kinesin or superior cervical ganglion 10 (SCG10), a microtubule binding protein in axons [20, 46]. However, in APLIP1 mutants, the *Drosophila* homologue of a JNK-interacting protein (JIP), transport of vesicles is reduced in both directions, but mitochondrial transport is reduced only in the retrograde direction, suggesting a unique regulatory feature of mitochondrial transport [47]. In addition, the alpha subunit of the heterotrimeric G protein G12 (Gα12) has been shown to stimulate JNK activity [48]. Notably, Gα12 is targeted to mitochondria and itself affects mitochondrial motility [49]. These studies suggest that the JNK pathway exerts some specific effects on axonal transport of mitochondria.

Could the ROS effects on mitochondrial transport be mediated via changes in Ca^2+^ levels? We show that excess ROS do increase Ca^2+^ levels (Fig 4A and 4B) and that the ROS-impaired mitochondrial mobility is rescued by Ca^2+^ chelation (Fig 4I–4L), consistent with ROS acting on mitochondrial transport via Ca^2+^. It is notable that H_2_O_2_-induced Ca^2+^ levels are rescued to physiological levels by the extracellular Ca^2+^ chelator EGTA (Fig 4I and 4J), suggesting that this Ca^2+^ increase results from Ca^2+^ influx from the extracellular environment. Several Ca^2+^ channels on the plasma membrane are regulated by redox status [34]. For example, H_2_O_2_ induces Ca^2+^ influx through L-type or T-type voltage-dependent Ca^2+^ channels (VDCCs) [50, 51]. ROS are also known to stimulate Ca^2+^ influx through channels involved in receptor-induced Ca^2+^ signals such as transient receptor potential (TRP) channels and store-operated Ca^2+^ channels (SOC), mediated by Orai channel proteins [52, 53]. Thus, we propose that elevated Ca^2+^ levels are induced by ROS through activation of Ca^2+^ channels on the plasma membrane. In addition, while most studies focus on the effects of elevated Ca^2+^ levels on mitochondrial transport [15–17, 54, 55], experimentally decreased intracellular Ca^2+^ levels reduce mitochondrial transport (Fig 5), suggesting that optimal Ca^2+^ levels are important for mitochondrial transport. This might explain a previous study showing that while the impaired mitochondrial transport caused by MPP^+^ can be rescued by the thio-antioxidant N-acetylcystein (NAC), it is not rescued by a high concentration of EGTA (2.5 mM), probably capable of reducing Ca^2+^ levels below physiological levels [56].

Although Ca^2+^ regulates mitochondrial transport, ROS-impaired mitochondrial transport is not fully rescued by reducing Ca^2+^ (Fig 4A–4D), suggesting that other factors might be involved in ROS regulation of mitochondrial transport. Indeed, several signaling pathways have been implicated in the regulation of axonal transport. Axonal mitochondria are recruited in response to Nerve Growth Factor (NGF), and this recruitment may be regulated via phosphoinositide 3-kinase (PI3K) or MAPK signaling [18]. Glycogen synthase kinase 3 (GSK3) can phosphorylate kinesin light chain (Klc) to inhibit axonal transport, while activation of AKT/GSK3β stimulates axonal mitochondrial transport [19, 21]. Finally, activation of the JNK pathway phosphorylates Khc, reducing the interaction between kinesins and microtubules and inhibiting anterograde axonal transport [20]. Here we show that activation of the JNK pathway reduces anterograde mitochondrial transport, and these defects are rescued by specific knockdowns (Fig 6A–6C). This anterograde regulation might result from the phosphorylation of kinesin by JNK [20]. In the retrograde direction, knockdown of the JNK pathway decreases mitochondrial transport (Fig 6A–6C). Since JIPs are required for stress-induced JNK activation [57–59], and mutation of a *Drosophila* JIP homologue reduces mitochondrial transport only in the retrograde direction [47], it is likely that disruption of the JNK pathway also produces retrograde-specific defects of mitochondrial transport.

How might Ca^2+^ and the JNK pathway interact in the ROS regulation of mitochondrial transport? Some studies suggest that elevated Ca^2+^ levels activate the JNK pathway, and subsequently induce apoptosis or neurodegeneration [60–62]. However, it is likely that the JNK pathway is not simply downstream of Ca^2+^ signaling in ROS regulation of mitochondrial transport. ROS could increase Ca^2+^ influx by activating Ca^2+^ channels [50–53], and activate the JNK pathway via apoptosis signal-regulating kinase (ASK1) [6, 36, 63], providing parallel, largely independent pathways of ROS-activated Ca^2+^ influx and JNK signaling. Activation or down-regulation of JNK signaling does not affect Ca^2+^ levels (S3 Fig), supporting the parallel-effect hypothesis. In addition, Ca^2+^ inhibits mitochondrial transport in both directions [15–17], but activation of JNK signaling inhibits mitochondrial transport anterogradely (Fig 6). Since Ca^2+^ influx and the JNK pathway could be induced by ROS via parallel pathways and produce different effects on mitochondrial transport, it is likely that they regulate mitochondrial transport in parallel.

In addition to elucidating mechanistic connections between ROS and mitochondrial transport, our studies provide evidence that mitochondrial quality control is related to transport. Excess ROS and abnormal Ca^2+^ homeostasis not only impair mitochondrial transport (Figs 1, 3, 4 and 5) but also reduce mitochondrial length (Fig 7). In addition, when mitochondrial transport is rescued (Figs 1, 3, 4K and 4L), mitochondrial lengths are also rescued (Fig 7). Previous studies have shown that mutations of Mitofusin reduce mitochondrial length, impair mitochondrial transport and increase Ca^2+^ levels in axons [22, 23, 39], consistent with our findings, and together reinforcing that mitochondrial fission-fusion are highly interrelated. Moreover, both mitochondrial membrane potential and transport are reduced in response to ROS (Figs 3 and 7C), suggesting the association between mitochondrial metabolic activity and transport. This idea is supported by studies showing that some neurodegenerative disease models, such as reduced frataxin expression or PINK1 mutation [12, 13], show reduction of both mitochondrial transport and membrane potential.

Due to the complicated interrelation in mitochondrial transport, morphology, and quality control, ROS-induced defects of mitochondrial transport would not be the only factor leading to neurodegeneration. ROS-induced deficits in mitochondrial motility are partially recovered after ROS insult, but the axonal degeneration is irreversible [25], supporting the hypothesis that instead of one factor, axonal degeneration could be caused by the combination of defects of mitochondrial transport, the imbalance of fission/fusion and metabolic state.

In conclusion, we provide evidence that ROS specifically impairs the axonal transport of mitochondria. This ROS-impaired mitochondrial transport is caused by an imbalance of Ca^2+^ homeostasis and activation of the JNK pathway. We also show the close interrelationship of mitochondrial transport, fission-fusion balance, and metabolic state in response to ROS. Thus, this study provides probable mechanistic links between ROS and mitochondrial transport, and suggests a new role for ROS in the induction of neurodegeneration.

## Materials and methods

### *Drosophila* strains

*w;+;D42-Gal4*, *UAS-mitoGFP* (Dr. Pallanck, University of Washington) and *w;sp/Cyo;D42-Gal4*, *UAS-ANF-GFP* (Dr. Saxton, UC Santa Cruz) were used to visualize GFP signals in mitochondria and dense core vesicles (DCVs), respectively, in motor neurons. *UAS-SOD1* and *UAS-SOD2* from the Bloomington Stock Center were crossed with *w;+;D42-Gal4*, *UAS-mitoGFP* to generate *UAS-SOD1; D42-Gal4*, *UAS-mitoGFP* and *UAS-SOD1; D42-Gal4*, *UAS-mitoGFP*. We used these two strains to perform the rescue experiments in Figs 1, 2 and 4. To examine calcium (Ca^2+^) levels in motor neurons, we received fly strains *UAS-GCaMP6m* from the Bloomington Stock Center, and generated *UAS-GCaMP6m/+; D42-Gal4/+*. To activate or down-regulate the JNK pathway, we used *UAS-Hep*^*B2*^ (Bloomington Stock Center) and *UAS-Bsk RNAi* (VDRC), respectively. These fly strains were also crossed with the control strain, *w;+;D42-Gal4*, *UAS-mitoGFP*, to generate *UAS-Hep*^*B2*^; *D42-Gal4*, *UAS-mitoGFP* and *UAS-Bsk RNAi; D42-Gal4*, *UAS-mitoGFP*. All flies were maintained in normal fly food at 25°C with 40%-60% humidity and a 12 hr light/dark cycle.

### Preparation of primary neuronal cell culture and dissected larvae for live imaging

For *in vitro* cell culture, 4 brains and ventral ganglia were dissected from third-instar larvae in Schneider’s medium and were incubated in Ca^2+^/Mg^2+^ free saline (137mM NaCl, 2.7mM KCl, 0.36mM NaH_2_PO_4_·H_2_O, 11.9mM NaHCO_3_, 5.6 mM glucose) with 0.7 mg/ml collagenase for 1 hr at room temperature. Samples were centrifuged at 300 g for 3 min and washed once with Schneider’s medium. We triturated the tissue with siliconized pipette tips to disperse it into individual cells, which were then plated on concanavalin A-coated coverslips and incubated in Schneider’s medium containing 10% FBS in a humidified chamber at 25°C for 72 hrs. Oxidative stress was induced in primary cell cultures with 100 μM H_2_O_2_ treatment for 1 hr, followed by staining and imaging [25]. Mitochondria were imaged using the 60x oil-immersion objective lens of an upright laser scanning confocal microscope (LSCM, Nikon Eclipse 90i) with a 488 nm laser. To induce oxidative stress i*n vivo*, second to third instar larvae were starved for 3 hrs and then maintained on normal fly food containing 20 mM paraquat for 24 hrs [27]. To image mitochondria or DCVs *in vivo*, third instar larvae were prepared using previously described methods [13, 28, 64]. Briefly, third instar larvae were pinned and opened through the cuticle on the dorsal side in HL6 buffer with 4 mM L-glutamate. Fat bodies and muscles were removed exposing the intact nervous system, including ventral ganglia and segmental nerves. The dissected larva was moved to a glass slide and covered with a coverslip using double-sided tape as a spacer. Mitochondria or DCVs in segmental nerves were observed using the 60x oil-immersion objective lens of an upright time-lapse laser confocal microscope (LSCM, Nikon Eclipse 90i) with a 488 nm laser.

### Analysis of mitochondrial and DCV transport

Time lapse confocal images of mitochondrial transport were acquired in the middle (segment A4) of larval segmental nerves using LSCM at 1 s intervals for 200 s. For cell culture, the time resolution is 1 frame/s for 120 s. We used the *Manual Tracking* plug-in with ImageJ software to track individual organelle. The percentage of moving mitochondria was analyzed using the *Cell Counter* plug-in with Image J software for both *in vitro* and *in vivo* experiments. For *in vivo studies*, four additional parameters were analyzed: flux, velocity, duty cycle, and run lengths. Flux is defined as the number of mitochondria that cross an assigned point in one-minute interval. If the average velocity in one interval is larger than 0.1 μm/s or smaller than -0.1μm/s and the motion is sustained in at least three frames, we considered this a “run”. The percentage of moving mitochondria is the fraction of mitochondria that move either anterogradely or retrogradely. Velocity is defined as the average velocity in the total run. Duty cycle is defined as the percentage of time spent moving in a particular direction. Run length is the average distance moved per run [13, 28, 64]. All kymographs of mitochondrial transport were prepared using Nikon NIS-Elements software [13, 28]. For transport of DCVs, time-lapse images were acquired using LSCM with 1 frame/s for 120s *in vitro* or 200s *in vivo*. Velocity were analyzed from kymographs using MetaMorph software *in vitro*. For *in vivo* analysis of DCV transport, the *Manual Tracking* plug-in with ImageJ software was used to track individual DCVs.

### Measurement of Ca^2+^ levels

Neuronal cultures expressing GCaMP6 in motor neurons were washed and incubated in S2 saline (120 mM NaCl, 5 mM KCl, 8 mM MgCl_2_, 2 mM CaCl_2_, 10 mM HEPES). To chelate extracellular Ca^2+^ or both extracellular and intracellular Ca^2+^, cells were incubated in Ca^2+^ free saline (S2 saline without CaCl_2_) containing 0.1 mM EGTA or both 0.1 mM EGTA and 10 μM BAPTA-AM for 30 min, respectively [60, 65, 66]. To increase intracellular Ca^2+^ levels, cells were treated with S2 saline containing 10 μM ionomycin or 2 μM thapsigargin [65]. Mean intensity of GCaMP6 fluorescence was thresholded and measured in whole cells with Nikon NIS-Elements software. To examine the effects of Ca^2+^ levels on mitochondrial or DCV transport, the percentage of moving mitochondria was determined under conditions described above.

### Quantification of mitochondrial density and lengths

Mitochondrial density was determined by assessing the number of mitochondrial pixels contained in fixed lengths of segmental nerves using MetaMorph software. The second frame of the time lapse images was used to reduce background noise and sharpen images [28]. Then images were thresholded, binarized, and density was measured by obtaining the integrated intensities (total number) of white mitochondrial pixels within the nerve. Mitochondrial lengths were measured with Nikon NIS-Elements software. Only non-overlapping mitochondria in neural processes were selected for length measurements.

### Measurement of mitochondrial membrane potential

Mitochondrial membrane potential was measured with tetramethylrhodamine methyl ester (TMRM) [12, 13, 28, 41]. Cells were incubated in Schneider’s medium with 25 nM TMRM for 10 minutes, and then were imaged in medium with 6.25 nM TMRM to maintain dye equilibrium. The TMRM images were thresholded and the intensity of mitochondrial fluorescence (Fm) was measured with Nikon NIS-Elements software. The fluorescence intensity in cytosol (Fc) was determined in 5 pixel x 5 pixel regions next to the mitochondria. The mitochondrial membrane potential was then expressed as the intensity ratio of mitochondrial fluorescence (Fm) and cytosolic fluorescence (Fc) which is logarithmically related to the membrane potential.

### Statistics

All statistical analysis was performed using GraphPad Prism 5 software. All error bars in graphs indicate mean ± SEM. In the *in vivo* studies, the experimental unit is an individual larva. In the *in vitro* studies, the experimental unit is a cell. Data were analyzed using a one-way ANOVA with Bonferroni’s post-test for multiple group comparisons, paired or unpaired Student’s t-test for two group comparisons. In all cases, at least three independent experiments were performed.

## Supporting information

S1 Fig A time course treatment of H_2_O_2_ treatment increases mitochondrial ROS and reduces mitochondrial transport. (A) Mitochondrial roGFP ratio (405 nm/488 nm) increases after H_2_O_2_ treatment for 2 hrs. (B) The percentage of moving mitochondria is reduced after after H_2_O_2_ treatment for 1 hr. Error bars indicate mean ± SEM. Significance is determined by one-way ANOVA with Bonferroni’s post-test. *p<0.05, **p < 0.01, and ***p < 0.001.
(TIF)

S2 Fig BAPTA-AM only is not enough for both extracellular and intracellular Ca^2+^ chelation. Representative Ca^2+^ imaging with BAPTA and ionomycin treatment is measured by the intensity of GCaMP6 indicator. Treatment of BAPTA-AM and ionomycin dramatically increases the intensity of GCaMP6 indicator.
(TIF)

S3 Fig Activation or down-regulation of the JNK pathway does not affect intracellular Ca^2+^ levels. (A) Representative Ca^2+^ imaging with overexpression of Hep^B2^ or down-regulation of Bsk; Ca^2+^ is measured by the intensity of GCaMP6 fluorescence. Scale bars indicate 10 μm. (B) Quantitative results from (A). H_2_O_2_ treatment produces an increase of Ca^2+^ levels, but neither overexpression of Hep^B2^ nor down-regulation of Bsk produces a significant difference in the result. Error bars indicate mean ± SEM. Significance is determined by one-way ANOVA with Bonferroni’s post-test. **p < 0.01.
(TIF)

S4 Fig JNK signaling has mild effects on ROS-induced defects of mitochondrial length and membrane potential. (A) ROS treatment shows a decrease of mitochondrial length in the control or Bsk knockdown background. (B) ROS treatment shows a decrease of mitochondrial membrane potential. Neither overexpression nor knockdown of the JNK pathway affects mitochondrial membrane potential. Error bars indicate mean ± SEM. Significance is determined by one-way ANOVA with Bonferroni’s post-test. *p<0.05 and ***p < 0.001.
(TIF)
