# Supplementary figures and images for: ROS regulation of axonal mitochondrial transport is mediated by Ca2+ and JNK in Drosophila
Source: PLoS One. 2017 May 18;12(5):e0178105. doi: 10.1371/journal.pone.0178105 (PMC5436889; doi:10.1371/journal.pone.0178105)

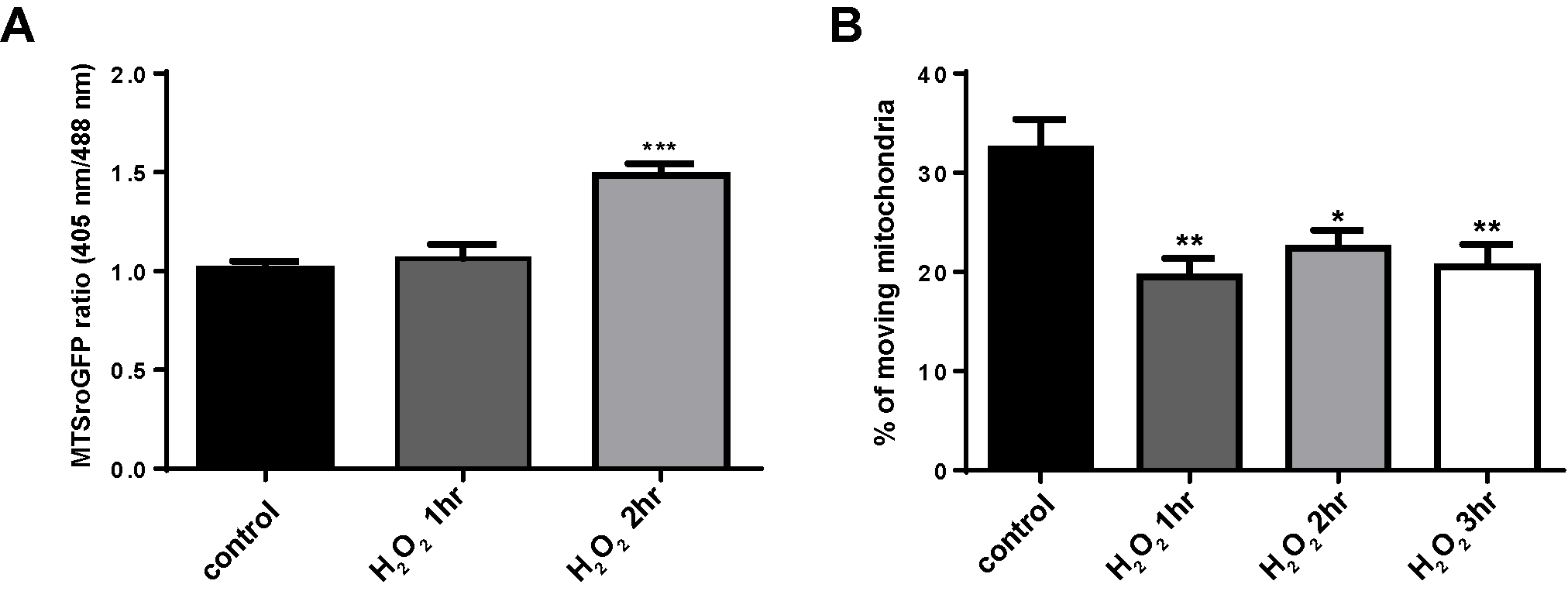

Supplement: S1 Fig — (A) Mitochondrial roGFP ratio (405 nm/488 nm) increases after H2O2 treatment for 2 hrs. (B) The percentage of moving mitochondria is reduced after after H2O2 treatment for 1 hr. Error bars indicate mean ± SEM. Significance is determined by one-way ANOVA with Bonferroni’s post-test. *p<0.05, **p < 0.01, and ***p < 0.001. (TIF) [file pone.0178105.s001.tif]

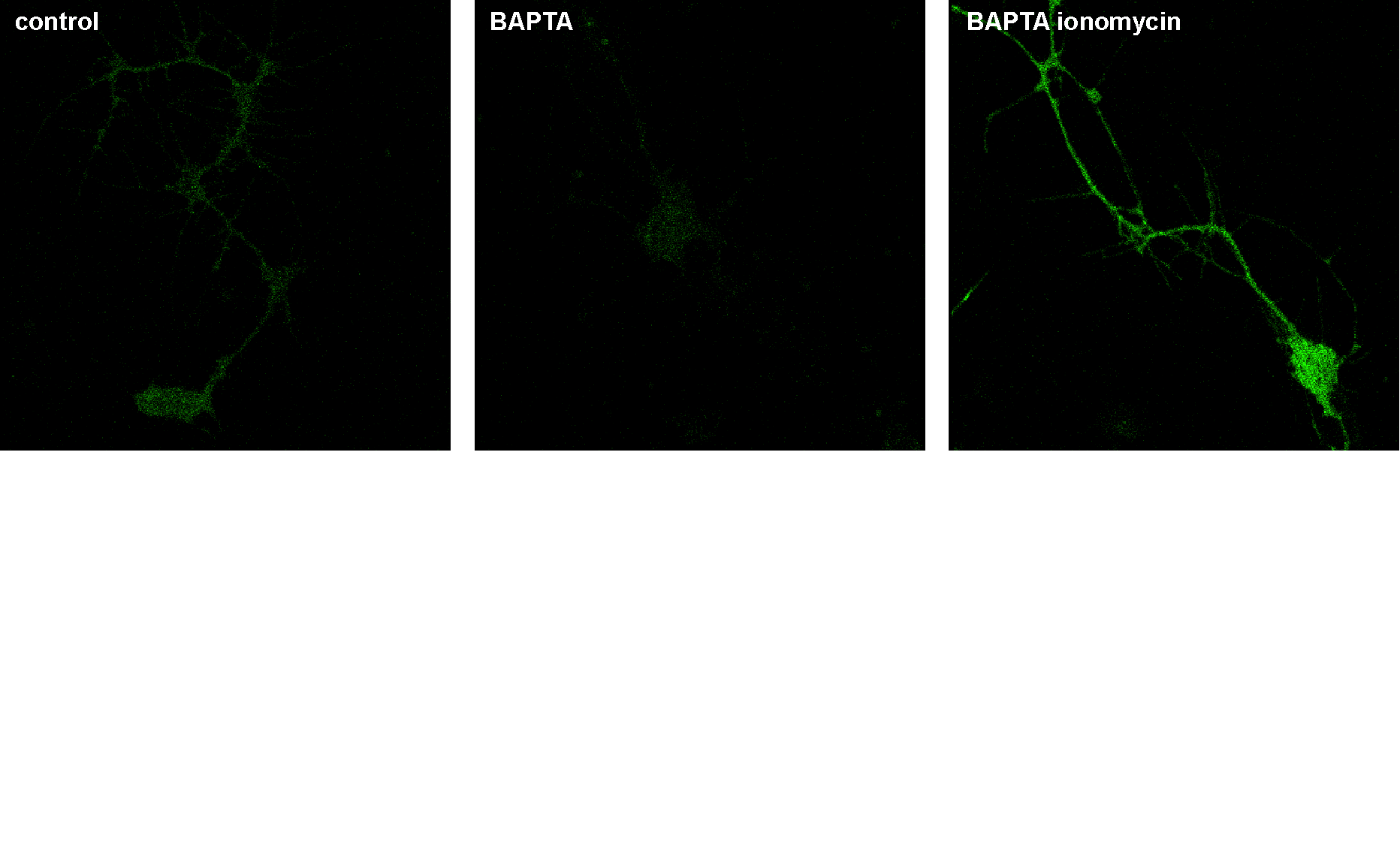

Supplement: S2 Fig — Representative Ca2+ imaging with BAPTA and ionomycin treatment is measured by the intensity of GCaMP6 indicator. Treatment of BAPTA-AM and ionomycin dramatically increases the intensity of GCaMP6 indicator. (TIF) [file pone.0178105.s002.tif]

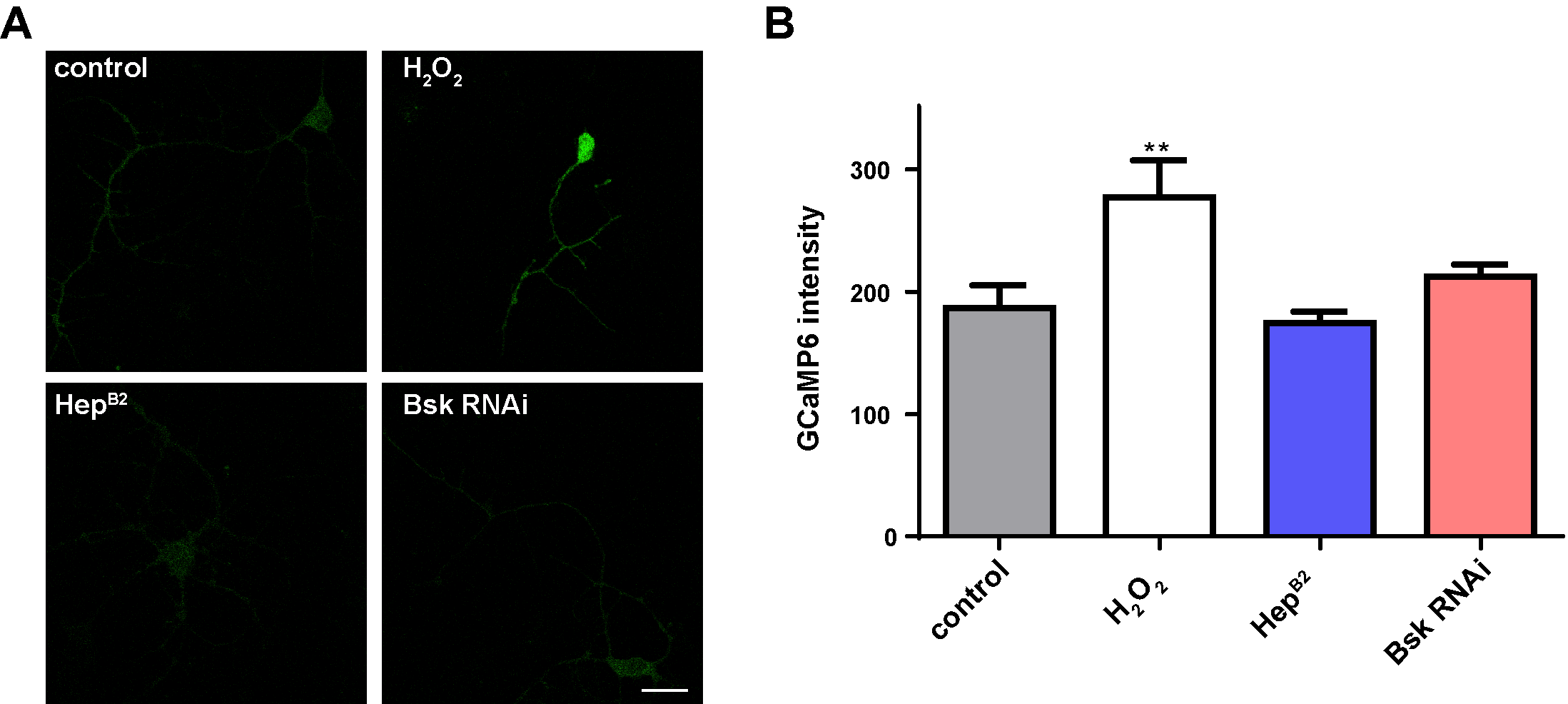

Supplement: S3 Fig — (A) Representative Ca2+ imaging with overexpression of HepB2 or down-regulation of Bsk; Ca2+ is measured by the intensity of GCaMP6 fluorescence. Scale bars indicate 10 μm. (B) Quantitative results from (A). H2O2 treatment produces an increase of Ca2+ levels, but neither overexpression of HepB2 nor down-regulation of Bsk produces a significant difference in the result. Error bars indicate mean ± SEM. Significance is determined by one-way ANOVA with Bonferroni’s post-test. **p < 0.01. (TIF) [file pone.0178105.s003.tif]

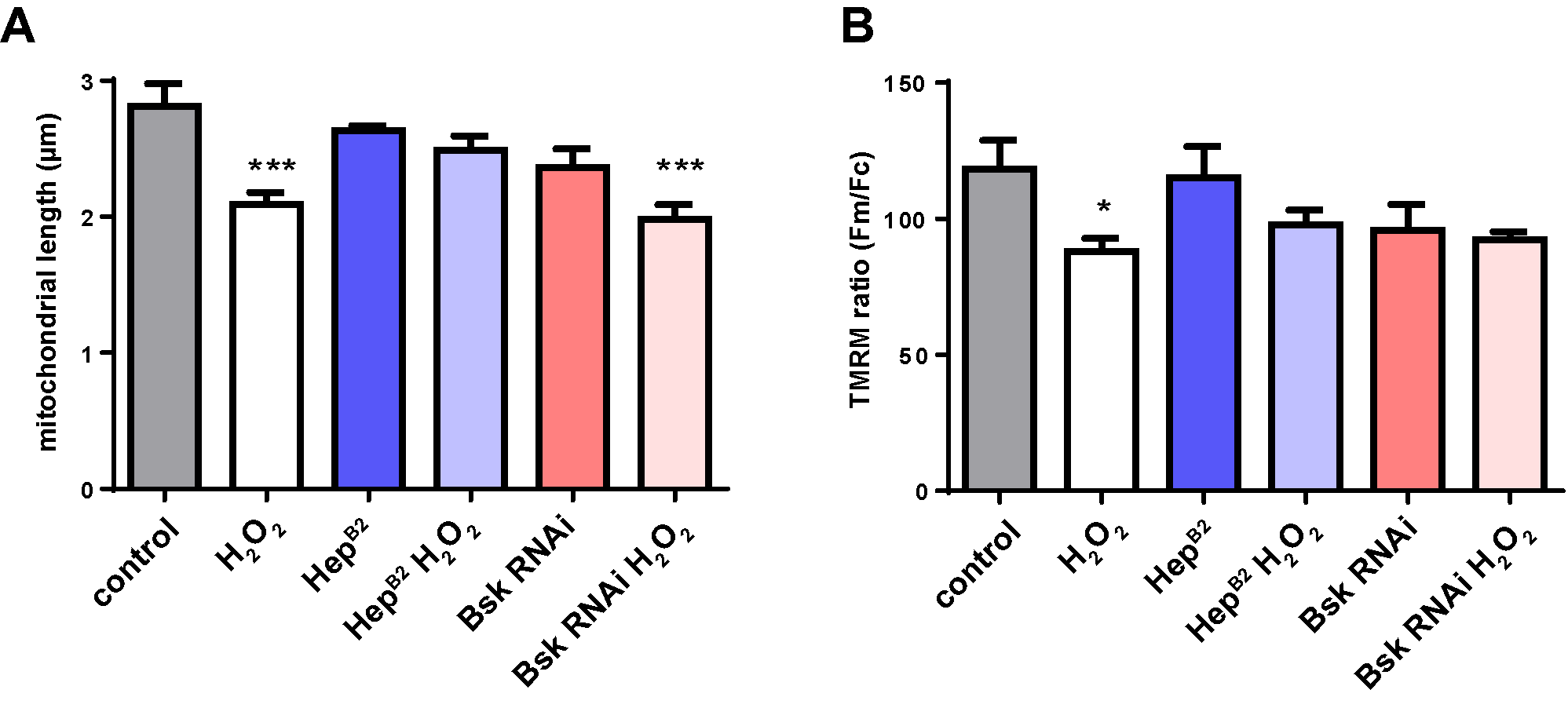

Supplement: S4 Fig — (A) ROS treatment shows a decrease of mitochondrial length in the control or Bsk knockdown background. (B) ROS treatment shows a decrease of mitochondrial membrane potential. Neither overexpression nor knockdown of the JNK pathway affects mitochondrial membrane potential. Error bars indicate mean ± SEM. Significance is determined by one-way ANOVA with Bonferroni’s post-test. *p<0.05 and ***p < 0.001. (TIF) [file pone.0178105.s004.tif]
